# Global Gene Expression Profiling of Endothelium Exposed to Heme Reveals an Organ-Specific Induction of Cytoprotective Enzymes in Sickle Cell Disease

**DOI:** 10.1371/journal.pone.0018399

**Published:** 2011-03-31

**Authors:** Samit Ghosh, Fang Tan, Tianwei Yu, Yuhua Li, Olufolake Adisa, Mario Mosunjac, Solomon F. Ofori-Acquah

**Affiliations:** 1 Aflac Cancer Center and Blood Disorders Service, Department of Pediatrics, Emory University School of Medicine, Atlanta, Georgia, United States of America; 2 Department of Biostatics and Bioinformatics, Emory University School of Medicine, Atlanta, Georgia, United States of America; 3 Winship Cancer Institute, Emory University School of Medicine, Atlanta, Georgia, United States of America; 4 Department of Pathology and Laboratory Medicine, Emory University School of Medicine, Atlanta, Georgia, United States of America; 5 Center for Endothelial Biology, Children's Healthcare of Atlanta, Atlanta, Georgia, United States of America; King Abdullah University of Science and Technology, Saudi Arabia

## Abstract

**Background:**

Sickle cell disease (SCD) is characterized by hemolysis, vaso-occlusion and ischemia reperfusion injury. These events cause endothelial dysfunction and vasculopathies in multiple systems. However, the lack of atherosclerotic lesions has led to the idea that there are adaptive mechanisms that protect the endothelium from major vascular insults in SCD patients. The molecular bases for this phenomenon are poorly defined. This study was designed to identify the global profile of genes induced by heme in the endothelium, and assess expression of the heme-inducible cytoprotective enzymes in major organs impacted by SCD.

**Methods and Findings:**

Total RNA isolated from heme-treated endothelial monolayers was screened with the Affymetrix U133 Plus 2.0 chip, and the microarray data analyzed using multiple bioinformatics software. Hierarchical cluster analysis of significantly differentially expressed genes successfully segregated heme and vehicle-treated endothelium. Validation studies showed that the induction of cytoprotective enzymes by heme was influenced by the origin of endothelial cells, the duration of treatment, as well as the magnitude of induction of individual enzymes. In agreement with these heterogeneities, we found that induction of two major Nrf2-regulated cytoprotective enzymes, heme oxygenase-1 and NAD(P)H:quinone oxidoreductase-1 is organ-specific in two transgenic mouse models of SCD. This data was confirmed in the endothelium of post-mortem lung tissues of SCD patients.

**Conclusions:**

Individual organ systems induce unique profiles of cytoprotective enzymes to neutralize heme in SCD. Understanding this heterogeneity may help to develop effective therapies to manage vasculopathies of individual systems.

## Introduction

Sickle cell disease (SCD) is a chronic inflammatory disease characterized by abnormally shaped red blood cells with a devastating impact on the endothelium [1]. A major clinical hallmark of the disease is episodes of painful vaso-occlusion, leading to ischemia/reperfusion injury, tissue hypoxia and organ damage [1]. Studies in transgenic SCD mice have demonstrated a cardinal role for adhesion molecules in the interaction of leukocytes and erythrocytes with the endothelium [2], [3], [4], [5], [6]. Antioxidants attenuate experimentally induced stasis in transgenic mice with SCD [2], [3], [4], while activation of the redox-sensitive transcription factor NF-κB is implicated in vascular inflammation [7]. Collectively, these findings suggest that reactive oxygen species (ROS) and oxidative intermediates contribute to the vaso-occlusive process in SCD.

Heme is arguably the major source of oxidative stress in SCD. Circulating blood contains virtually no detectable free heme as it is bound instantly with high affinity by plasma proteins, notably, hemopexin [8], [9]. The heme-hemopexin complex is transported to the liver and removed via a CD91-mediated endocytosis. However, it is estimated that approximately 30g of hemoglobin is released per day from hemolyzed erythrocytes in patients with SCD [10] with 30% of the total hemolysis being intravascular [11]. Thus, the plasma of patients with SCD contains excess cell-free hemoglobin and heme [10], [12] and is depleted of hemopexin [9]. The high plasma heme in these patients is due largely to oxidation of hemoglobin to ferric (Fe^3+^) methemoglobin, which in turn readily releases free heme into the circulation [13]. While sustained oxidant stress inevitably causes cell and tissue damage, and vascular injury [12], [14], [15], patients with SCD do not develop atherosclerotic disease. This relatively mild endothelial dysfunction is attributed partly to the activation of adaptive cytoprotective mechanisms in SCD patients. This idea is supported by the increased expression of heme oxygenase-1 (HO-1) in renal tissues, and in circulating endothelial cells of patients, as well as in multiple tissues of transgenic SCD mice [16], [17]. HO-1 is the inducible form of heme oxygenase, and it converts heme to carbon monoxide and biliverdin, byproducts with vasodilatory and cytoprotective properties [18], [19], [20]. Concurrent up-regulation of biliverdin reductase and p21 in mononuclear leukocytes is consistent with the enhanced expression of HO-1 in patients with SCD [21]. The vasculoprotective property of HO-1 is evident in other experimental disease models of ischemia-reperfusion [22], atherogenesis [23] and vascular constriction [24]. However, the scope of cytoprotection in SCD has not previously been defined, and recent studies in transgenic SCD mice indicate that the level of induction of HO-1 alone is insufficient to neutralize the oxidative burden of the disease [25], [26]. We hypothesized that transcriptional profiling of endothelial cells exposed to heme would provide a means to dissect the full extent of the endothelium's capacity to neutralize heme, and provide a non biased approach to identifying cytoprotective enzymes in SCD.

## Materials and Methods

### Ethics Statement

Studies on post-mortem human tissues were approved by the Institutional Review Board of Emory University and Grady Memorial Hospital and written informed consents obtained from next of kin (protocol 00008845). Experiments using mice was approved by the Institutional Animal Care and Use Committee (IACUC) of Emory University (protocol 113-2007).

### Materials

Hemin (Sigma-Aldrich, St. Louis, MA) was dissolved in 0.25M NaOH solution, pH was adjusted to 7.6 with HCl and the solution was then filter-sterilized before treating endothelial cells. Primary antibody against HO-1 was obtained from Stressgen (Assay Design Inc, Ann Arbor, Michigan). Primary antibodies for NAD(P)H quinone oxidoreductase 1(NQO1), and β-actin used in immunoblot experiments were purchased from Santa-Cruz Biotech Inc (Santa Cruz, CA). Primary antibodies for NQO1 and von Willebrand factor (vWF) used in immunohistochemistry were procured from Abcam Inc (Cambridge, MA). Horse radish peroxidase (HRP) conjugated secondary antibodies used included anti-mouse, anti-goat and anti-rabbit IgG (Santa Cruz Biotech Inc).

### Primary endothelial cell cultures

The following primary human endothelial cells: pulmonary artery (PAECs), pulmonary microvascular (PMVECs) and dermal microvascular (DMVECs) were purchased from Lonza Inc (Allendale, NJ), while brain microvascular endothelial cells (BMVECs) were purchased from Cell System Corp. (Kirkland, WA). PAECs, PMVECs and DMVECs were cultured at 37°C and 5% CO_2_ in a humidified incubator in EGM-2 endothelial media (Lonza), supplemented with growth factors and 5% fetal bovine serum, while BMVECs were cultured with CSC complete Medium (Cell System Corp) in culture dishes precoated with attachment factor (Cell System Corp). These cells were fed at 48-h intervals and required remarkably strict attention for *in vitro* culture. PAECs, PMVECs, DMVECs and BMVECs were treated with a concentration range (0–25 µM) of freshly prepared hemin. Cells were replenished with fresh medium containing hemin every 48 hours for 1–7 days, and harvested for analysis. Endothelial cell cultures of passage 3–5 were used in all experiments.

### RNA isolation, hybridization and microarray analysis

Total RNA was isolated from endothelial cells using the Trizol Reagent (Invitrogen, Carlsbad, CA), treated with 2.5 µl of DNase I, purified on a Qiagen Cleanup column (Qiagen, Valencia, CA) and eluted in 20 µl of RNase-free water. Ribosomal RNA was depleted using RiboMinus Human/Mouse Transcriptome Isolation Kit (Invitrogen). RNA quality and yield was assessed on an Agilent Bioanalyzer 2100 (Agilent Technologies, Palo Alto, CA). Sense strand expression analysis of RNA was performed using the Affymetrix GeneChip Whole Transcript (WT) Sense Target Labeling Assay protocol (Affymetrix). cDNA was synthesized using the GeneChip WT cDNA Synthesis and Amplification Kit (Affymetrix) as directed by the manufacturer as template for *in vitro* transcription (IVT) amplification with T7 RNA Polymerase to produce multiple copies of cRNAs. In the second cycle of cDNA synthesis, random primers were used in reverse transcription to convert the cRNA into single-stranded DNA. Single-stranded cDNA was fragmented and end-labeled with the GeneChip WT Terminal Labeling Kit (Affymetrix). Labeled single-stranded DNA (5.5 µg) was hybridized to the Affymetrix GeneChip Human U133 Plus 2.0 Array, stained on a GeneChip Fluidics Station 450 and scanned on a GeneChip Scanner 3000 7G (Affymetrix). The Affymetrix Expression Console was used to record probe cell intensity files (.CEL) and probe level summarization files (.CHP) generated. All microarray data is MIAME compliant and has been deposited in NCBI's Gene Expression Omnibus (GEO) with accession number GSE25014 and can be accessed at http://www.ncbi.nlm.nih.gov/geo/query/acc.cgi?acc=GSE25014.

### Validation of microarray data in cultured endothelial cells

Low Density Arrays were custom designed by Applied Biosystems. Real-Time PCR was performed on an optical plate in a 10-µl reaction volume containing 10 ng of cDNA per reaction with TaqMan Gene Expression Master Mix (Applied Biosystems). Sequences were amplified using the Applied Biosystems 7900HT Sequence Detection System with the PCR profile: 50°C for 2 min, 95°C for 10 min, followed by 45 cycles at 95°C for 15 s, and 60°C for 1 min. Samples were tested in duplicate, in parallel with four housekeeping genes (18S, ACTB, GAPDH, GUSB) in the low-density array assays, or in parallel with a single housekeeping gene (GUSB) in analysis of single gene targets. Gene expression data were normalized relative to the geometric mean of the four housekeeping genes (18S, ACTB, GAPDH, GUSB) for the low-density array studies. Raw data were obtained by using SDS2.3 software (Applied Biosystems), and Real-Time StatMinerTM software (Integromics) used to perform a quality control for all runs and to determine *ΔΔCt* values as previously described [27].

### Transgenic mice with SCD

A colony of the Berkeley knock-out homozygous sickle mice developed by Paszty et al.[28], is maintained in our institution. The recently described knock-in transgenic mice with SCD were provided by Dr. Townes [29]. Mouse genotypes were confirmed either by hemoglobin gel electrophoresis or by PCR.

### Analysis of NQO1 and HO-1 gene expression in mouse tissues

Organs were isolated whole, immediately immersed in liquid nitrogen, and total RNA extracted using RNeasy kits (Qiagen) and reverse-transcribed with a High-Capacity cDNA Archive Kit (Applied Biosystems). We carried out the relative quantitative real-time PCRs following TaqMan Applied Biosystems protocols. We tested samples in triplicate in parallel with the housekeeping gene GUSB. We used StatMiner software (Integromics) to perform quality controls for all runs and relative quantification *ΔΔCt* analyses to calculate the fold differences as previously described [27], between AA vs AS and AA vs SS mouse samples.

### Immunoblot

Endothelial cells seeded in 100-mm culture dishes were rinsed with ice cold phosphate buffered saline (PBS), supplemented with 1% complete protease inhibitor cocktail (PIC)(Roche, Indianapolis, IN) and 1 mM PMSF. Lysates were prepared with 200 µl of ice-cold Cell Lysis Buffer (Cell Signaling Technology, Beverly, MA) containing 1% triton X-100 (v/v) supplemented with fresh 1 mM PMSF and 1% PIC and incubated on ice for 30 min. To prepare tissue homogenates, organs from mice were harvested and snap-frozen in liquid nitrogen. The frozen tissues were homogenized in ice-cold RIPA buffer (Cell Signaling Technology, Beverly, MA) containing 1% complete PIC, 1% phosphatase inhibitor cocktail (Sigma) and 1 mM PMSF. Homogenates were clarified by centrifugation at 14,000 rpm for 15 min at 4^o^C. The supernatant soluble proteins were quantified using a Lowry protein assay (Sigma, St Louis, MO). Thirty micrograms of cellular protein or 50 µg homogenate of tissue protein was combined with 2X Laemmli buffer (Sigma, St Louis), boiled for 5 min and resolved by electrophoresis on a 10% polyacrylamide gel (BioRad). Samples were blotted onto nitrocellulose membranes, probed with appropriate antibodies, and protein bands were identified by chemiluminescence and quantified using a Fujifilm LAS-3000 plus quantitative imaging system (FujiFilm, Valhalla, NY).

### Immunohistochemistry

Sections (5 µm) of formalin fixed paraffin embedded (FFPE) tissues were de-paraffinized, rehydrated and processed for antigen retrieval using Dako Antigen Retrieval Solution. Tissue peroxidases were inactivated by treatment with 3% H_2_O_2_ for 10 min, and the sections pre-treated with antibody diluent solution containing 1% BSA, followed by an overnight incubation at 4°C with primary antibodies for NQO1 (10 µg/ml), HO-1 (10 µg/ml) or vWF (5 µg/ml). Labeling was accomplished with biotinylated secondary antibodies and streptavidin-HRP using Biotinylated Link Antibody kit (Dako North America Inc, Carpinteria, CA), AEC substrate chromogen, and counterstained with hematoxylin. Sections were mounted with aqueous media, examined using Olympus AX70 microscope and images were recorded with camera (Olympus U-CMAD3 DP70) and software (Olympus DP70/DP30 BW, ver.02.0201.147). Semi-quantitative histological scores based on a scale of 0-3, where 0  =  no stain, and 3 =  most intense staining, was performed by an experienced pathologist blinded.

### Statistical analysis

Microarray summarization files were analyzed using the Bioconductor in the R framework. [30] Two methods were used to generate gene-level expression index: the Li-Wong dChip model [31] and the RMA. [32] Genes assigned as "absent" call in all samples were excluded. The unpaired student's t-test was used on a gene-by-gene basis to test for differential expression between hemin and vehicle treated endothelial cells. The p-values were adjusted to false-discovery rate (FDR) [33]. Differentially expressed genes were selected based on an FDR cutoff of 0.2, and a fold-change cutoff of 1.25. Genes identified as differentially expressed using both dChip and RMA results were taken as the final selection. Differences in relative quantities of mRNA and protein expression were analyzed using unpaired student's t*-*test with two-tailed distribution, with the GraphPad Prism Software (version 5.0).

## Results

### Gene expression profile of endothelial cells treated with hemin

**Figure 1:**
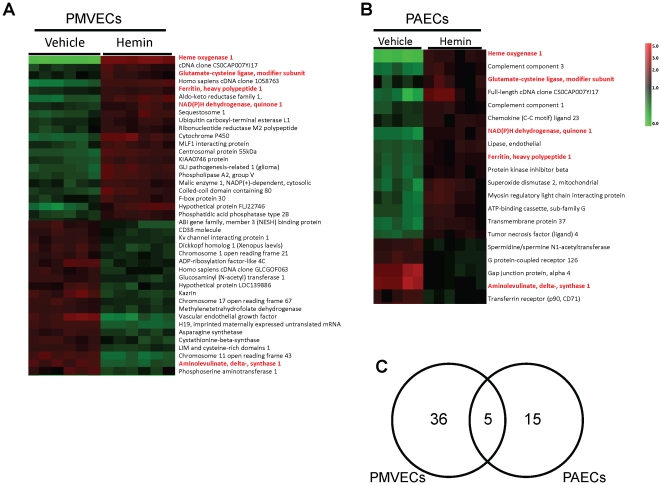
Heme regulated genes in endothelial cells. Hierarchical cluster analysis of differentially expressed genes in primary lung endothelial cells, PMVECs (A) and PAECs (B) successfully segregates cultures treated with vehicle from those treated with 5 µM hemin. The unpaired student's t-test was used on a gene-by-gene basis to test for differential expression between hemin and vehicle-treated cultures. (C) Venn diagram showing the number of genes differentially regulated by hemin in PMVECs and PAECs.

**Figure 2:**
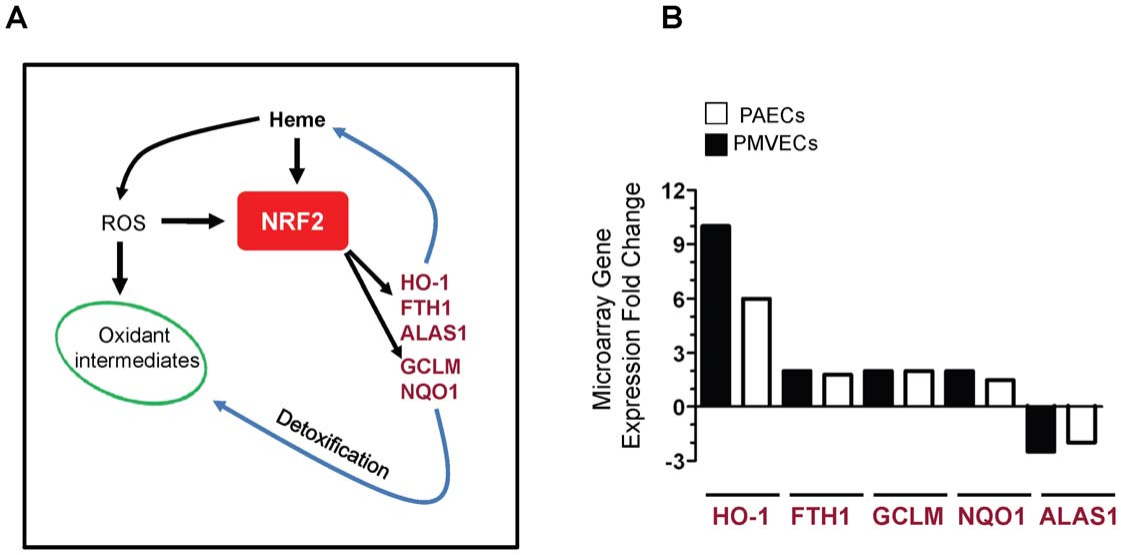
Induction of Nrf-2 regulated genes by hemin. (A) Central role of Nrf2 in the response of the endothelium to heme. Genes whose expression was altered by hemin in both PAECs and PMVECs are shown. ROS =  reactive oxygen species. (B) Microarray data of differentially expressed genes regulated by Nrf2 in PAECs and PMVECs treated with hemin. Data shown is mean fold change in gene expression as arbitrary units relative to the level of expression by control cells (n = 5).

In preliminary studies we determined that relatively low (1–5 µM) concentrations of hemin were not toxic to endothelial cells in culture (data not shown). Thus, PAECs and PMVECs were treated with freshly prepared hemin (5 µM) every 48 hours for 7 days, RNA was isolated from these cultures followed by microarray analysis. Differentially expressed genes successfully segregated endothelial cell cultures treated with hemin from those treated with vehicle in hierarchical cluster analysis (Figure 1A, B). Forty-one and twenty genes, respectively, were significantly differentially regulated by hemin in PMVECs and PAECs (Table 1 and Table 2). Among this list were three genes which are already known to be regulated by heme, HMOX1 (*NM_002133*), which codes for HO-1, and genes for ferritin heavy chain (*AA083483*) and delta aminolevulinate synthase 1 (*NM_000688*). This finding provided validation for the experimental, statistical and bio-informatics approach we used in this study. Two additional genes glutamate-cysteine ligase modifier (GCLM; *NM_002061*) and NADPH:quinone oxidoreductase-1 (NQO1; *NM_000903*), were significantly induced by hemin in both PAECs and PMVECs. Thus a total of 5 genes were induced in both endothelial cell types and 51 genes were uniquely induced in either PAECs or PMVECs (Figure 1C). Ingenuity Pathway analysis revealed that all the 5 genes induced by hemin in both endothelial cell types are regulated by the redox-sensitive master transcription factor NF-E2 related factor 2 (Nrf-2). They include gene products, such as HO-1 and FTH1 that act directly on heme, and others (e.g. NQO1 and GCLM), that detoxify oxidative intermediates generated by heme (Figure 2A). The level of induction of these cytoprotective genes was variable in both PMVECs and PAECs, and ranged from 1.3 to 10 fold (Figure 2B).

**Table 1:** Genes differentially regulated by hemin in PMVECs.

Probeset ID	Accession	Gene Name	Fold change
203665_at	NM_002133	heme oxygenase (decycling) 1	10.72
234986_at	AA630626	Full-length cDNA clone CS0CAP007YJ17	2.37
236140_at/	NM_002061	glutamate-cysteine ligase, modifier subunit	2.25
1555854_at	AA594609	*Homo sapiens* cDNA clone 1058763	2.13
200748_s_at/214211_at	NM_002032	ferritin, heavy polypeptide 1	1.29
209699_x_at/	U05598	aldo-keto reductase family 1, member C2	1.81
210519_s_at/	NM_000903	NAD(P)H dehydrogenase, quinone 1	1.77
244804_at/	AW293441	Sequestosome 1	1.55
201387_s_at	NM_004181	ubiquitin carboxyl-terminal esterase L1	1.49
201890_at/	BE966236	ribonucleotide reductase M2 polypeptide	1.46
205749_at	NM_000499	cytochrome P450, family 1	1.46
218883_s_at	NM_024629	MLF1 interacting protein	1.45
218542_at	NM_018131	centrosomal protein 55 kDa	1.43
212311_at/	AA522514	KIAA0746 protein	1.42
204222_s_at/	NM_006851	GLI pathogenesis-related 1 (glioma)	1.42
215870_s_at	AL158172	phospholipase A2, group V	1.39
204058_at	AL049699	Malic enzyme 1, NADP(+)-dependent,	1.34
225241_at	AA570507	coiled-coil domain containing 80	1.32
226541_at	AI808182	F-box protein 30	1.31
220637_at	NM_024785	hypothetical protein FLJ22746	1.28
212226_s_at	AA628586	phosphatidic acid phosphatase type 2B	0.78
223395_at	AB056106	ABI gene family, member 3	0.78
205692_s_at	NM_001775	CD38 molecule	0.76
221307_at	NM_014592	Kv channel interacting protein 1	0.76
204602_at	NM_012242	dickkopf homolog 1 (Xenopus laevis)	0.76
223126_s_at/	AI159874	chromosome 1 open reading frame 21	0.73
202207_at	BG435404	ADP-ribosylation factor-like 4C	0.73
238720_at	AV661099	*Homo sapiens* cDNA clone GLCGOF063	0.72
239761_at	AI088120	glucosaminyl (N-acetyl) transferase 1, core 2	0.72
228654_at	AU145277	hypothetical protein LOC139886	0.71
213478_at	AB028949	kazrin	0.71
236863_at	BF592860	chromosome 17 open reading frame 67	0.70
201761_at	NM_006636	methylenetetrahydrofolate dehydrogenase	0.69
210512_s_at	AF022375	vascular endothelial growth factor	0.68
224997_x_at/224646_x_at	AL575306	H19, imprinted maternally expressed untranslated mRNA	0.57
205047_s_at	NM_001673	asparagine synthetase	0.66
1553972_a_at	BC007257	cystathionine-beta-synthase	0.63
218574_s_at	NM_014583	LIM and cysteine-rich domains 1	0.63
202409_at	X07868	chromosome 11 open reading frame 43	0.57
205633_s_at	NM_000688	aminolevulinate, delta-, synthase 1	0.53
223062_s_at	BC004863	phosphoserine aminotransferase 1	0.37

**Table 2:** Genes differentially regulated by hemin in PAECs.

Probeset ID	Accession	Gene Name	Fold Change
203665_at	NM_002133	heme oxygenase (decycling) 1	5.23
217767_at	NM_000064	complement component 3 /// similar to Complement C3 precursor	2.11
236140_at/	NM_002061	glutamate-cysteine ligase, modifier subunit	1.98
234986_at	AA630626	Full-length cDNA clone CS0CAP007YJ17 of Thymus of Homo sapiens (human)	1.92
1555229_a_at	BC007010	complement component 1, s subcomponent	1.81
210548_at/210549_s_at	U58913	chemokine (C-C motif) ligand 23	1.72
201468_s_at/	NM_000903	NAD(P)H dehydrogenase, quinone 1	1.57
219181_at	NM_006033	lipase, endothelial	1.53
214211_at	AA083483	ferritin, heavy polypeptide 1	1.51
223551_at	AF225513	protein kinase (cAMP-dependent, catalytic) inhibitor beta	1.43
215223_s_at	W46388	superoxide dismutase 2, mitochondrial	1.37
228097_at	AW292746	myosin regulatory light chain interacting protein	1.36
204567_s_at	NM_004915	ATP-binding cassette, sub-family G (WHITE), member 1	1.33
1554485_s_at	BI825302	transmembrane protein 37	1.32
207426_s_at	NM_003326	tumor necrosis factor (ligand) superfamily, member 4 (tax-transcriptionally activated glycoprotein 1, 34kDa)	1.29
213988_s_at	BE971383	spermidine/spermine N1-acetyltransferase	0.8
213094_at	AL033377	G protein-coupled receptor 126	0.76
40687_at	M96789	gap junction protein, alpha 4, 37 kDa (connexin 37)	0.75
205633_s_at	NM_000688	aminolevulinate, delta-, synthase 1	0.7
207332_s_at/208691_at	NM_003234	transferrin receptor (p90, CD71)	0.46

**Figure 3:**
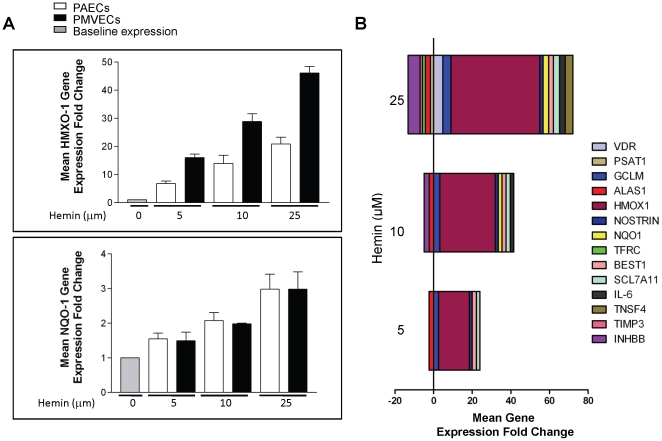
Validation of microarray data by qRT-PCR. (A) Total RNA from PAECs and PMVECs treated with hemin (0–25 µM) was analyzed for the expression of HO-1 and NQO1. Data shown is mean fold change relative to the vehicle (0 µM hemin) +/− SD for three independent experiments each in triplicate (n = 9). (B) Low-density array data showing changes in expression of fourteen genes in PMVECs treated with a concentration range (0–25 µM) of hemin for 7 days. Note the concentration-dependent increase in the number of genes altered by hemin (n = 12).

**Figure 4:**
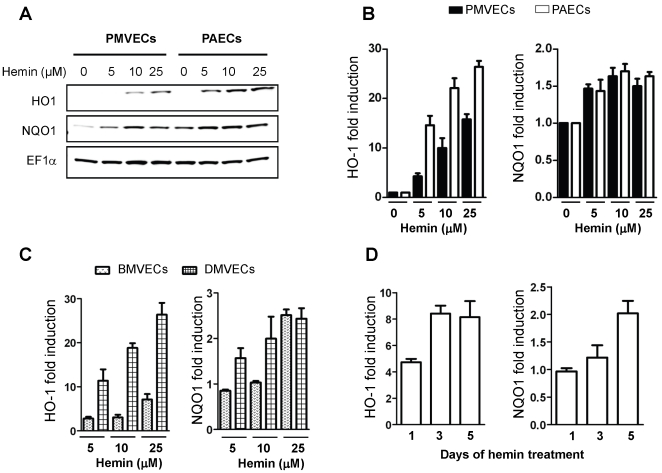
Concentration- and time-dependent induction of HO-1 and NQO1 by hemin in endothelial cells. (A) Western blot analysis confirming concentration dependent induction of HO-1 and NQO1 in PMVECs and PAECs treated with hemin for 7 days. Blots were probed for EF-1αto control protein loading. (B, C) Quantification of HO-1 and NQO1 protein expression in PAECs, PMVECs, BMVECs and DMVECs treated with hemin and vehicle, assayed by western blot analysis. Data shown is mean fold change in protein level as arbitrary units relative to the EF-1α-normalized expression in vehicle treated cells (n = 9). (D) Quantification of western blot showing variable timing of HO-1 and NQO1 induction by hemin (5 µM) in PAECs (n = 9).

To validate the microarray data, cultures of PAECs and PMVECs were treated with a concentration range of hemin (0-25 µM) for 1–7 days. Total RNA was extracted and mRNA expression analyzed using a multiplex low-density array qRT-PCR platform, or single-gene qRT-PCR assay. The fold increase in HO-1 and NQO1 mRNA due to 5 µM hemin as determined by qRT-PCR in the validation experiments was similar to the expression level identified from the initial microarray analysis (Figure 3A). NQO1 mRNA level was virtually identical in PAECs and PMVECs at all hemin concentrations studied. HO-1 mRNA was induced by over 40-fold in PMVECs at the highest concentration tested. The number of genes impacted by hemin, and the magnitude of fold-change was dose-dependent, as shown for a selection of 14 genes, which were positively or negatively regulated by hemin (Figure 3B). Next, we determined whether hemin-induced alterations in HO-1 and NQO1 mRNA level were reflected at the protein level. For cultures treated with hemin for 7 days, both HO-1 and NQO1 proteins were induced in a concentration dependent manner (Figure 4A). Induction of HO-1 was markedly more robust than NQO1 in both PMVECs and PAECs, while the level of HO-1 protein was consistently higher in PAECs than in PMVECs (Figure 4B). To determine the relevance of these findings in other vascular beds, endothelial cells derived from the brain (BMVECs) and skin (DMVECs) were studied. Hemin strongly induced expression of HO-1 protein in DMVECs, while the induction of HO-1 in BMVECs and of NQO1 in both cell types was modest (Figure 4C). HO-1 induction occurred within 24 hours of treatment, while significant induction of NQO1 occurred after 5-days of treatment (Figure 4D and data not shown). While these validation studies confirmed the data from our microarray results, they showed that the induction of HO-1 and NQO1 by hemin is influenced by the duration of treatment and the origin of the endothelial cells.

### Organ-specific expression of HO-1 and NQO1 in transgenic SCD mice

**Figure 5:**
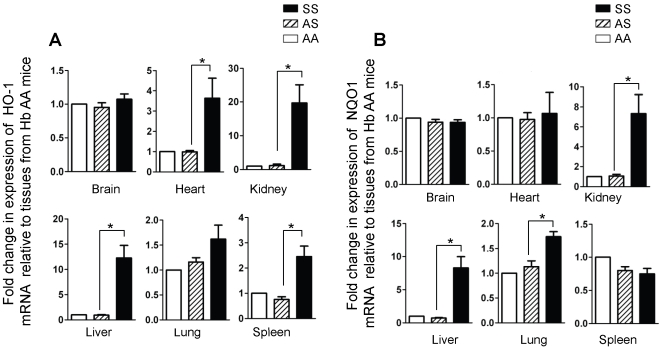
Organ-specific induction of HO-1 and NQO1 in SS mice. Total RNA was isolated from the indicated organs from transgenic mice of the Townes model, expressing normal human hemoglobin (Hb AA n = 4), or with sickle trait (Hb AS n = 7) or SCD (Hb SS, n = 6). Expression of HO-1 (A) and NQO1 (B) was determined by relative quantitative real-time PCR. Data shown are the mean ± SD. *p<0.05.

To investigate the relevance of our microarray data in the context of SCD, transgenic mice expressing exclusively sickle hemoglobin (Townes SS or Berkeley sickle), and their corresponding controls (AS, AA, hemi) were studied. Using the *ΔΔCt* values for samples from control AA mice, we calculated the relative quantities of HO-1 and NQO1 mRNA for the various organs from AS and SS mice. We found that HO-1 transcripts in the kidneys and liver were over 10-fold higher in the SS mouse compared to the level in control AS littermate, or AA mice, while the level in the SCD heart and spleen were 3-4 fold higher compared to the controls (Figure 5A). The difference in HO-1 mRNA level in SS, AS and AA lungs did not reach statistical significance, while no differences were found for this transcript in the brains of SS, AS and AA mice (Figure 5A). These findings were confirmed in organs isolated from the Berkeley sickle and hemizygote mice (data not shown). The pattern of induction of NQO1 in the SCD mice was different from that observed for HO-1. In particular, NQO1 mRNA level was significantly higher in the SCD mouse lungs of both the Townes and Berkeley models while the relative quantity in the SCD mouse hearts were similar to the level in control AS and AA mice (Figure 5B and data not shown).

**Figure 6:**
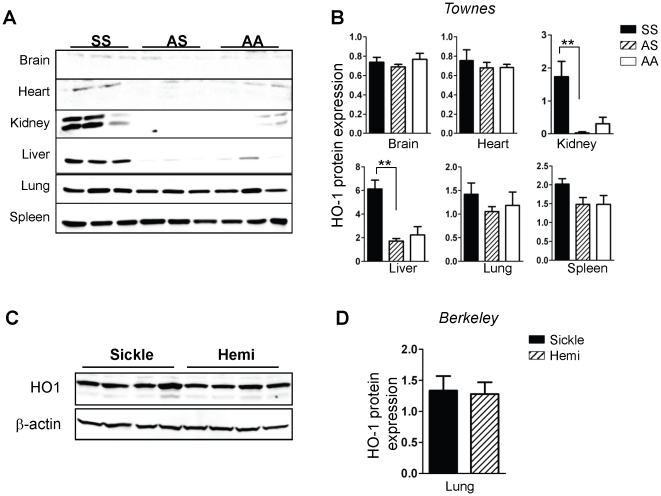
Heterogeneity of enhanced HO-1 expression in SS mice. (A) Western blot analysis, of snap-frozen organs isolated from Townes SS, AS and AA mice, for HO-1 expression. (B) Quantitative data of western blot experiments. Data shown are mean arbitrary units of β-actin-normalized HO-1 expression in the indicated organs and genotypes of Townes mice (n = 6). (C) Western blot analysis of HO-1 in whole lungs of Berkeley sickle mice and control hemizygotes. (D) Quantitative data of western blot experiments for HO-1 in the Berkeley mice (n = 4). **p<0.01.

**Figure 7:**
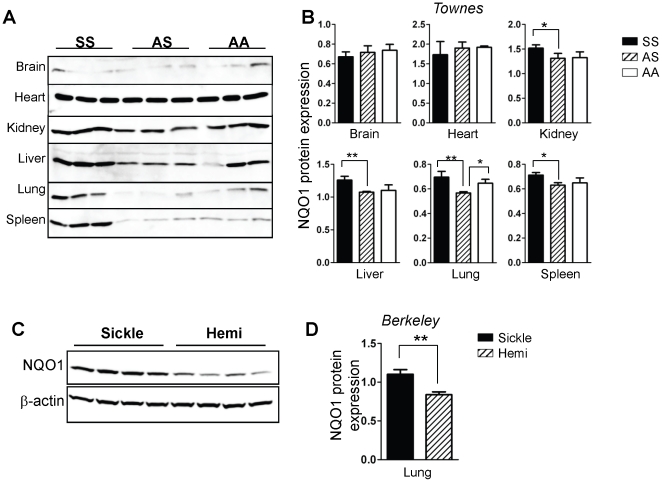
Enhanced expression of NQO1 in SS mice. (A) Western blot analysis for NQO1 expression in snap-frozen organs from Townes SS, AS and AA mice. (B) Quantitative data of western blot experiments. Data shown are mean arbitrary units of β-actin-normalized NQO1 expression in the indicated organs and genotypes (n = 6). (C) Western blot analysis of NQO1 in whole lungs of Berkeley sickle mice and control hemizygotes. (D) Quantitative data for NQO1 protein in the Berkeley mice lungs showing arbitrary units of β-actin-normalized expression (n = 4). *p<0.05, **p<0.01.

Since, mRNA levels do not always correspond to protein expression, tissues were examined by immunoblots using identical conditions to permit rigorous comparisons of the amount of individual enzymes in tissues from the same mice, and allow comparisons across genotypes. There was low-to-undetectable HO-1 expression in the brain and heart in mice of all genotypes, while abundant expression was evident in the SS kidney and liver, and in the spleen and lungs of mice of all genotypes (Figure 6A). Quantification of the data in Figure 6A confirmed that HO-1 expression in the SS kidney and liver is significantly elevated, on the contrary, expression in the lung was only modestly higher than in control AS and AA mice (Figure 6B). Moreover, there was no difference in lung HO-1 expression in the Berkeley sickle and hemizygote mice (Figure 6C-D). There was increased expression of NQO1 in the SS mice kidney, liver, lung and spleen, but not in the brain or heart (Figure 7A), and this interpretation was confirmed by quantitative analysis (Figure 7B). The result in the lung was confirmed in the Berkeley model (Figure 7C–D). Collectively, our data in two mouse models of SCD demonstrate a highly variable induction of two major cytoprotective enzymes (HO-1 and NQO1) in major organs impacted by SCD for the first time.

### Expression of HO-1 and NQO1 in lung endothelium of SCD patients

**Figure 8:**
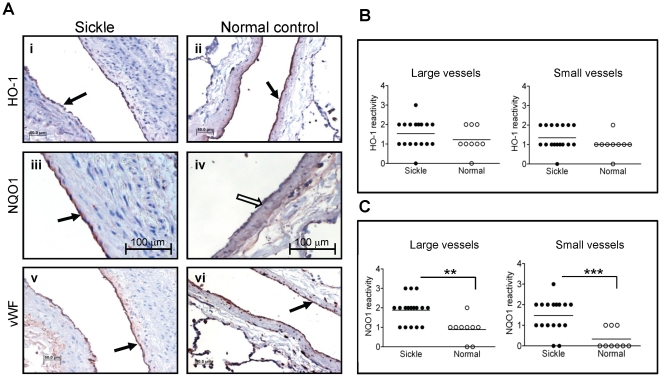
Expression of HO-1 and NQO1 in lung endothelium of SCD patients. Representative histological images (A) showing expression of HO-1 (i-ii), NQO1 (iii-iv) and vWF (v-vi) in the endothelium of post-mortem lung tissues of SCD patients and normal control. Note comparable intensities of HO-1 staining in both SCD and control endothelium (solid arrow). Robust staining of NQO1 in the endothelium of the SCD tissue (iii; solid arrow) compared to a relatively weak staining in the normal tissue (iv; open arrow). vWF staining of consecutive sections of both tissues demonstrates an intact endothelium in the NQO1 negative control tissue. Histological scores of HO-1 (B) and NQO1 (C) staining (SCD patients n = 17, normal controls n = 9). **p<0.01 and ***p<0.001.

Results showing that HO-1 expression was not elevated in the lungs of two murine models of SCD were unexpected and contrary to a general assumption supported by our own results in other organs, that HO-1 expression is upregulated in SCD. The lung pathology in SCD mice is relatively mild compared to the more severe phenotype in humans [34]. Therefore, we considered whether the HO-1 expression in the lung was reflective of a relatively low oxidative stress below the threshold for activating Nrf2. Alternatively, our data represented a hitherto unrecognized tissue diversity in HO-1 expression in SCD. To resolve this issue, we studied HO-1 in post-mortem lung tissues from an archive at Grady hospital in Atlanta [35]. We found comparable intensities of HO-1 staining in the endothelium of SCD (n = 17) and normal lung tissues (n = 9) (Figure 8A i-ii), consistent with our data in the mouse models. NQO1 staining in the endothelium of the same tissues was however markedly more intense in the SCD samples (Figure 8A iii-iv). Histological scoring using a semi-quantitative method by a pathologist unaware of the identity of individual specimens confirmed these observations (Figure 8B-C).

## Discussion

Cytoprotective enzymes offer a strategy for therapy of sickle vasculopathies, and they may also be involved in the wide clinical spectrum of SCD. Previous studies have examined HO-1 expression in mononuclear leukocytes and in selected tissues in patients and transgenic mice with SCD [16], [17], [21]. However, HO-1 expression in the endothelium in SCD, where plasma heme is likely to have the most impact had not previously been studied. Moreover, the cytoprotective profile of many organs impacted by SCD remained unknown. This study was designed to address these inter-related issues.

In conditions of oxidant stress, the transcription factor Nrf2 binds to antioxidant response elements to induce expression of both phase I (acute) and phase II (late-stage) detoxifying enzymes. Our microarray data showed that several enzymes belonging to both classes were induced in endothelial cells treated with hemin (Figure 1, 2 and Table 1, 2). Enzyme induction was heterogeneous with respect to duration of treatment with hemin, magnitude of gene expression and the vascular origin of the cells (Figure 3, 4 and data not shown). These *in vitro* findings were mirrored *in vivo* as expression of two major cytoprotective enzymes were variably elevated in different organs, in two murine models of SCD (Figure 5, 6, 7). The lack of significant upregulation of HO-1 in the lungs of SS mice was unexpected, and contrary to the findings from a previous study [16]. This discordance is likely due to the use of different control animals in this, and the previous study. Here, we compared HO-1 expression in SCD and control mice of identical genetic backgrounds, in both the Townes and Berkeley models (Figure 5, 6, 7). Our findings in the sickle mice were supported by results of HO-1 expression in human lung endothelium (Figure 8), and is pertinent to the lack of lung pathology in the only patient described thus far with HO-1 deficiency [36], [37], [38] as well as in mice with deletion of the HO-1 gene [39].

The lack of association between HO-1 activity or expression and specific clinical phenotype in SCD patients is a major limitation of our understanding of the role of this cytoprotective enzyme in SCD. Nonetheless, it is reasonable to assume that HO-1 exerts some influence on this disease including in the lung given its indirect effects via carbon monoxide and biliverdin. For instance, HO-1 null mice are rescued from death from ischemic lung injury by the administration of carbon monoxide [40]. A sleeping beauty gene construct delivered to the liver, and inhaled carbon monoxide improve vascular stasis in the skin of SCD mice [16], [26]. Unlike these distant effects, however, the degradation of heme requires direct HO-1 enzymatic action, particularly during episodes of acute hemolysis. Our results highlight a relatively blunted capacity for this direct function in the brain and lung in SCD mice suggesting that these organs may be more vulnerable to hemolytic crises. However, our data in postmortem lung endothelium does not provide a comprehensive account of HO-1 expression in the lung of SCD patients. We therefore caution against generalizations of our findings in the context of the whole lung in SCD patients. This prudence is supported by the results of a pilot study showing there is wide variation of HO-1 mRNA level in a small number of FFPE lung tissues of SCD patients (data not shown). Unfortunately, FFPE tissues from SCD patients are not readily available, and moreover, they are difficult to analyze due to the degradation of nucleic acids by formalin and long periods of storage. Thus, an alternative approach is required to assess whether HO-1 expression is variably regulated in different compartments of the same tissue, and by factors that are independent of tissue type.

The potential benefits of NQO1 in SCD is likely evident in adults, given the late induction of this enzyme in endothelial cells treated with hemin (Figure 4D). Indeed, we found that NQO1 is not induced in young SS mice aged 4-8 weeks (data not shown). On the contrary, expression is markedly increased in lungs of adult SS mouse aged 13–27 weeks (Figure 7A–D), presumably timed to combat the increasing burden of oxidant stress in adults. We tested this idea and found that cytosolic fractions of adult SS mouse lungs reduce 2,6-dichlorophenol-indophenol (DCPIP), a potent oxidant, in an NQO-1 dependent manner, while fractions from young SS mouse lungs failed to reduce DCPIP (data not shown). NQO1 directly scavenges superoxide anion [41], [42], this property may be critical in the sickle vasculature because of the high oxidant burden of the disease, and the relatively low expression of super oxide dismutase in endothelium [42]. Finally, NQO1 may play a broader role in redox protection in SCD to prevent a variety of malignancies that develop in NQO1-null mice exposed to high oxidative burdens [43], [44], [45]. Naturally occurring polymorphisms that reduce NQO1 expression are associated with higher rates of solid tumors [46], [47], [48], poor survival in breast cancer [49] and carotid artery plaques in type II diabetes [50]. Therefore, the putative benefits of NQO1 in SCD may be subject to variation in a manner analogous to the variable NQO1 expression in the postmortem SCD lung tissues (Figure 8C).

We focused this study on HO-1 and NQO1 and therefore recognize the limitation imposed by this strategy in not examining further the expression of the three other Nrf2-regulated genes (ALAS1, GCLM and FTH1) identified in our micro array. Moreover, we did not investigate expression of HO-1 and NQO1 in other tissues in SCD patients in this study. We have begun to address the latter limitation in a follow-up study by measuring HO-1 level in the plasma of SCD patients. Preliminary results from that study show that plasma HO-1 level is markedly variable, and it correlates positively with multiple markers of endothelial activation and injury in SCD patients. These initial findings are consistent with the conclusion of the current study that upregulation of HO-1 expression in SCD is context dependent.

In conclusion, our study provides evidence that major organs impacted by SCD have uniquely different cytoprotective phenotypes in mouse models, with the brain apparently refractory to the oxidant stress of SCD. Expression of HO-1 in SCD patients requires further studies, including evaluating the role of endothelial dysfunction, inflammation and genetic heterogeneity in this process. NQO1 is induced in multiple organs in SCD, and may play a multifunctional protective role, most likely at later stages of the disease. The molecular heterogeneity of the response to hemolytic/oxidative stress defined in this study may help to develop more effective cytoprotective/antioxidant therapy for SCD.
